# Nonclonal Chromosome Aberrations and Genome Chaos in Somatic and Germ Cells from Patients and Survivors of Hodgkin Lymphoma

**DOI:** 10.3390/genes10010037

**Published:** 2019-01-10

**Authors:** Sara Frias, Sandra Ramos, Consuelo Salas, Bertha Molina, Silvia Sánchez, Roberto Rivera-Luna

**Affiliations:** 1 Laboratorio de Citogenética, Instituto Nacional de Pediatría, Cd. De Mexico, P.O. Box 04530, Mexico; sera_ramos@yahoo.com.mx (S.R.); bertha_molina@yahoo.com.mx (B.M.); sanchezsilvia2000@yahoo.com.mx (S.S.); 2 Instituto de Investigaciones Biomédicas, Universidad Nacional Autónoma de Mexico, Cd. De Mexico, P.O. Box 04510, Mexico; 3 Laboratorio de Genética y Cáncer, Instituto Nacional de Pediatría, Cd. De Mexico, P.O. Box 04530, Mexico; consusa@hotmail.com; 4 Subdirección de Hemato-Oncología, Instituto Nacional de Pediatría, Cd. De Mexico, P.O. Box 04530, Mexico; riveraluna@yahoo.com

**Keywords:** chromosome instability (CIN), chromoplexy, genome chaos, chromosomal heterogeneity, karyotype heterogeneity, nonclonal chromosome aberration (NCCA), second cancer, virus reactivation

## Abstract

Anticancer regimens for Hodgkin lymphoma (HL) patients include highly genotoxic drugs that have been very successful in killing tumor cells and providing a 90% disease-free survival at five years. However, some of these treatments do not have a specific cell target, damaging both cancerous and normal cells. Thus, HL survivors have a high risk of developing new primary cancers, both hematologic and solid tumors, which have been related to treatment. Several studies have shown that after treatment, HL patients and survivors present persistent chromosomal instability, including nonclonal chromosomal aberrations. The frequency and type of chromosomal abnormalities appear to depend on the type of therapy and the cell type examined. For example, MOPP chemotherapy affects hematopoietic and germ stem cells leading to long-term genotoxic effects and azoospermia, while ABVD chemotherapy affects transiently sperm cells, with most of the patients showing recovery of spermatogenesis. Both regimens have long-term effects in somatic cells, presenting nonclonal chromosomal aberrations and genomic chaos in a fraction of noncancerous cells. This is a source of karyotypic heterogeneity that could eventually generate a more stable population acquiring clonal chromosomal aberrations and leading towards the development of a new cancer.

## 1. Introduction

The population of cancer survivors in the world is continuously growing. In 2016, in the United States there was a population of survivors of more than 15 million people, and it is projected that by 2026 there will be more than 20 million in this country alone [1]. This is due to several factors, including that the population is growing and aging, that there is better and earlier detection of cancer, and, importantly, to the great success and effectiveness of anticancer therapies.

One of the cancers with a high likelihood of cure is Hodgkin’s lymphoma (HL), which is a neoplasm affecting the B cells of the lymphoid system and has average annual age-adjusted incidence rates of 3.2, 2.5, 2.3, and 1.3 per 100,000 in Whites, Blacks, Hispanics, and Asians, respectively. However, the major variation in the incidence is by the age at diagnosis, with more than a half of the cases occurring during the teenage years or early 20s [2,3]. Among adolescents, HL is the third most frequent cancer, preceded only by brain/CNS cancer and leukemia [1].

The survival rate for HL patients is near 90% at five years [1]. However, this high survival rate is associated with secondary events to treatment. In fact, survivors of all types of childhood cancers have a higher risk for subsequent hospitalization and spend five times as many days as compared with healthy individuals of similar age. In particular, HL survivors are among those with the highest risk of presenting with a new cancer and the highest number of days of hospitalization. In addition, approximately half of these days spent in the hospital are due to recurrences, but also to new primary cancers [4]. These long-term complications are likely due to the use of strong genotoxic agents as part of the anticancer treatment.

The purpose of this review is to provide a brief overview of the available data related on the genotoxic consequences of the aggressive and genotoxic anticancer treatment used in HL patients. The focus is on therapy-induced chromosomal abnormalities that have been considered as partially responsible for the azoospermia and oligospermia in male patients, as well as the development of new neoplasms that are observed in 10%–20% of the HL survivors.

## 2. Hodgkin Lymphoma Outline

HL is a lymphoproliferative malignancy of B cell origin. HL patients are classified in two entities, classical HL (cHL), comprising 95% of cases and the majority of histological types, and an uncommon second entity called nodular lymphocyte-predominant HL (NLP-HL). The incidence is variable, according with the region, 4.4–6.4 cases per 100,000 people in underdeveloped countries [5], while in the United States the incidence is 2.6 cases per 100,000 people and represents 11% of all lymphomas. HL is more frequent in young people between 20–40 years presenting a second peak at 60 years or older and affects males more frequently than females [2].

Two characteristic cell types can be found in cHL affected lymph nodes of (a) mononuclear Hodgkin (H) cells and (b) large multinucleated cells called Reed–Sternberg (RS). Hodgkin and Reed Sternberg cells (HRS) are pathognomonic for cHL and are intermixed in a cellular environment of non-neoplastic inflammatory cells [6]. HRS originate from defective, pre-apoptotic germinal center B cells, these cells are positive for the CD30 and CD15 surface markers and show constitutive activation of the NF-κB and JAK/STAT signaling pathways, which are essential for their survival. Positive regulation of these pathways results from mutation or inactivation of their negative regulators, including NFKBIA, NFKBIE, TNFAIP3, or gene amplification of its activators, such as REL, MAP3K14, JAK2, PD-L1, PD-L2, and JMJD2C [2]. HRS cells present chromosomal alterations common in other malignancies, such as deletions del(4q), del(6q), del(7q), or del(13q) and translocations such as t(2;15), t(14;18), or t(14;19). Microarray technology has evidenced the gain of genes, previously known to be constitutively expressed in cHL, like *STAT6* (12q31), *NOTCH1* (9q34), *JUNB* (19p13) and, recently, *TCF3* or *E2a* (19p13.3), associated also with pre-B cell acute lymphoblastic leukemia. Loss of heterozygosity analysis revealed that 80% of primary cHL cases displayed monoallelic losses of 16q21-q23, 6q25 (78%), 12p12 (75%), 3q26 (67%), and 2p23 (57%), however the implicated genes remain to be studied [7,8].

The precise etiology of HL is unknown, HL behaves as a multifactorial entity, presenting genetic and environmental risk factors. Genetic susceptibility has been evidenced by the existence of family aggregation. The study of families with two or more affected members with HL has allowed the detection of genes predisposing to HL. Rotunno et al. in 2016 [9] studied, by whole exome sequencing, 65 families with recurrent HL and found in two families, the only recurrent mutation found until now, a nonsynonymous c.3193G>A change in the *KDR* gene (kinase insert domain receptor) also known as VEGFR2 (vascular endothelial growth factor receptor 2), since most of the identified variants are “private” for each affected family. In addition, twin studies have shown that the risk for HL is 100 times higher in identical twins than in fraternal twins, indicating that in these families, the genetic component is stronger than environmental factors [2,9].

Recognized environmental risk factors involved in HL include the presence of the γ-herpes virus, autoimmune disease and immunosuppression. A high percentage ~40–90% of HL patients are positive for Epstein Bar Virus (EBV). Although the involvement of viral infection in the pathogenesis of HL is controversial, certain studies have shown that the activity of some EBV proteins contributes to the development and maintenance of HRS tumor cells. EBV virus may be in lytic or latent state; the lytic infection produces a large quantity of virions that kill the host cell, whereas the latent infection produces a reduced amount of viral proteins that retain the virus as an episome or integrated into the chromosomes, this latent state keeps the host cell alive and has been associated to cell growth and transformation through activation of different latent membrane proteins LMP1, LMP2A, and LMP2B, as well as EBNA1, EBER RNAs, and BART microRNAs. Functional studies of LMP1 and LMP2A have shown that the first activates NF-κB, Jun N-terminal kinase (JNK), and p38 mitogen-activated protein kinase pathways and the latter participates in the inhibition of apoptosis and evasion of the immune response. EBNA1 and LMP1 promote genomic instability, a well-known requirement for malignant transformation and microRNAs participate in immune evasion [2,8]. 

HL cells show telomere dysfunction; in EBV positive HL patients LMP1 viral protein induces inhibition and dysfunction of TRF2 (shelterins group) leading to telomere shortening in HL lymph nodes. Short telomeres induce chromosomal abnormalities, promoting telomere fusion which generate dicentric chromosomes, breakage-fusion-bridge cycles, abnormal chromosomal segregation, aneuploidy, and nonclonal structural chromosomal aberrations; all of these abnormalities are present in HRS cells [10]. Peripheral blood lymphocytes in HL patients also present telomere erosion. M’kacher et al. [11] showed that telomeric length was significantly shorter in HL patients without therapy as compared with healthy donors (8.3 vs. 11.7 kb length); five years after receiving Chemotherapy (CT), telomeres decreased in length but not significantly (7.64 kb length), while HL patients in complete remission recover their telomeric length (9.7 kb), suggesting that telomere length may be a risk factor for the occurrence of secondary cancers and diseases in long-term survivors [11].

## 3. Genotoxicity of the Anticancer Treatment in Hodgkin Lymphoma

Chemotherapy. In general, the treatment strategy for HL consists of a combination of CT and radiotherapy (RT). There are several CT regimens that include a mixture of agents that are efficient in killing cancer cells, in recent decades, the most used regimens are MOPP (Mechlorethamine, Oncovin, Procarbazine, and Prednisone), NOVP (Novantrone, Oncovin, Vinblastine, and Prednisone), COPP (Cyclophosphamide, Oncovin, Procarbazine, and Prednisone), and ABVD (Adriamycin, Bleomycin, Vinblastine, and Dacarbazine). These treatments include cytotoxic and genotoxic chemicals that affect tumor cells by damaging the DNA and interfering with the processes of DNA replication and/or repair or altering the processes of chromosome segregation during cell division (Table 1) [12,13].

In the 1960s, the first effective CT for HL was the MOPP regimen that included alkylating agents such as nitrogen mustard and procarbazine, which are recognized as potent clastogenic and mutagenic agents [14,15]. This regimen was effective in the treatment of advanced HL with or without radiation therapy, with a 65–70% survival five years after treatment; however, it had high reproductive toxicity and great carcinogenic potential. Since the 70s, several modifications to the MOPP regimen were introduced to maintain chemotherapeutic efficacy and reduce associated toxic effects. New schemes such as ABVD, NOVP, or mixtures of MOPP/ABVD were developed to avoid high doses of alkylating agents, produce fewer side effects, lower the incidence of secondary cancers and achieve an excellent recovery of reproductive function, providing adequate elimination of tumor cells and a disease-free survival at five years greater than 85% [16].

Currently there are chemotherapeutic schemes combining different components, number of cycles and improved radiation techniques (Supplementary Table S1) [17,18]. Moreover, targeted drugs and several monoclonal antibodies against targets in the HRS cells and immunoreactive cells in the tumor surrounding microenvironment have been developed, i.e., antibodies against CD30 antigen, a member of the TNF cell receptor superfamily is a promising treatment for increasing effectiveness and decreasing toxicity side effects (Supplementary Table S2) [17,19].

Radiotherapy. Ionizing radiation from several sources is used for RT, among patients treated for HL a strong correlation has been observed between the dose of RT and the radiation field size with the development of secondary solid tumors; the risk increases with larger RT fields, mainly for breast and lung tumors and non-Hodgkin lymphoma [20], for this reason, over the time, RT tends to use lower doses and smaller fields. During 1960–1990, the total dose used was 40–44 Gy applied after CT [21,22]. From 1995 to present, a combination of short cycles of CT with a total dose of 30-36 Gy has been applied to involved fields. From 2008 to present, thanks to better imaging and advancements in radiation delivery techniques, an innovative RT has been developed specifically directed to involved nodes (not fields) using 20–30 Gy. It is expected that this novel approach will reduce the volume of normal tissue receiving high doses of radiation and consequently might reduce the risk of second malignancies [23,24,25].

At the cellular level, ionizing radiation acts directly breaking the DNA molecule generating single and double strand breaks, these DNA lesions are repaired mainly by the error-prone nonhomologous end joining (NHEJ) and may produce numerical and structural chromosomal damage including aneuploidies, dicentrics, rings, acentric fragments, chromatid aberrations, telomere shortening, inversions, and translocations, as well as a high frequency of sister chromatids exchanges and micronuclei [11,26,27]. RT-induced genomic damage can be observed not only in the irradiated cells, but also in cells located in close proximity (bystander effect) or farther from irradiated cells (abscopal effect), through intercellular communication and signaling pathways, inflammatory processes, and the immune response [20,28]. These effects may contribute to the induction of chromosomal abnormalities observed in lymphocytes from LH survivors up to 14 years after receiving RT, suggesting the existence of damage in hematopoietic progenitor cells located in bone marrow [29,30].

## 4. Risk for a New Cancer in Hodgkin Lymphoma Patients

Anticancer treatments do not have a specific cell target, damaging the genetic material of both normal and cancerous cells. Induced genetic damage may be lethal and nonlethal. When occurring in somatic cells such as blood cells, lethal damage can generate anemias or infections, while in germ cells it can produce oligospermia and transitory or permanent azoospermia. Importantly, nonlethal damage can also have serious consequences, with surviving cells carrying numerical and structural chromosomal damage that in somatic cells could causes secondary cancers related to treatment, and in germ cells could result in abortions or offspring with genetic affectations [31,32].

The therapeutic regimens used in HL patients, with multi-agent CT and RT, have resulted in a population of young people who due to the stress of genotoxic anticancer treatment, have a high risk of developing a new and different cancer [33,34]. The estimated risk of secondary cancer is 43.6% with a 40-year cumulative incidence [35]. There are three main types of new primary cancer in HL survivors: non-Hodgkin lymphoma is reported in 17% of patients, 25% leukemia in CT treated patients, and 58% of solid tumors in CT+RT treated HL patients. Breast, lung, and colorectal cancer are the most common solid malignancies after CT treatment of HL [34]. A strong correlation has been observed between the dose of RT and the radiation field size with the development of secondary solid tumors [35]. Associated with CT, alkylating agents have been related with the development of therapy-related myelodysplastic syndrome and acute myeloid leukemia [33,36].

## 5. Genomic Instability, Chromosome Instability and Genomic Chaos

Genomic instability is a condition in which the genomes of a specific tissue or organism are constantly generating genetic alterations, both at a small scale, such as changes of a single nucleotide, microsatellite instability or at large scale, at the chromosome level, then called chromosomal instability (CIN), which is characterized by an increased rate of karyotype variability in a given cell population, and cell to cell variability [37]. CIN may be intrinsic (constitutional), associated with germinal mutations in genes related to chromosomal segregation such as *BUBR1* and *BUB1B* cause mosaic variegated aneuploidy [38], *FANC* genes in Fanconi anemia, or *ATM* in Ataxia Telangiectasia [39,40] these syndromes display CIN at the cellular level and an increased risk of cancer [37,41]. However, these types of mutations are rare and do not explain most sporadic cancers. CIN may be also extrinsic, related to nongenetic factors such as CT or radiation exposure, virus infection, and some physiologic processes like inflammation or aging; these extrinsic mechanisms of CIN are frequently associated with sporadic cancer [37,41]. CIN originated by intrinsic or extrinsic mechanisms, have the common feature of generating heritable chromosomal variation, producing new chromosomal combinations that may drive toward cell adaptation and then evolution of cancer [37,41,42].

**Figure 1:**
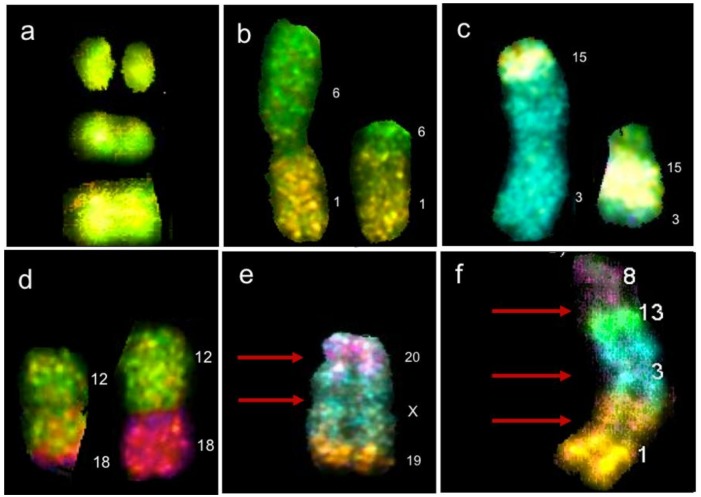
Chromosomal abnormalities found by multicolor fluorescence in situ hybridization (M-FISH) in lymphocytes of HL survivors. (**a**) Chromosome 12 with two double strand breaks (DSBs) in different arms of the same chromosome. (**b**) Balanced translocation t(1;6)(p?;p?). (**c**) Balanced translocation t(3;15)(p?;q?). (**d**) Balanced translocation t(12;18)(q?;p?); translocations in (b–d) resulting from erroneous DNA repair of two DSBs occurring on two nonhomologous chromosomes. (**e**) Rearrangement dicentric + deletion + translocation, resulting from four DSBs, two on the same chromosome X, one on chromosome 20 and one on chromosome 19. (d) Complex rearrangement resulting from multiple DSBs on multiple chromosomes, found in a cell with chaotic karyotype (chromoplexy). Red arrows represent centromeres, numbers represent the chromosomes involved in the rearrangement [48].

CIN can manifest as numerical CIN, consisting of gains or losses of whole chromosomes and structural CIN characterized by chromosomal rearrangements such as deletions, duplications, translocations, isochromosomes, dicentrics, complex rearrangements, massive rearrangement of the genome or genome chaos, and others [37,43]. Structural CIN can occur due to template switching or by erroneously repaired double strand breaks (DSBs) in the DNA [44]. The greater the number of DSBs, the more frequent and more complex structural chromosomal alterations are originated (Supplementary Table S3, Figure 1). Both numerical and structural CIN can be found in the same cell population and coexist in a single cell [45], as nonclonal chromosomal alterations (NCCA) present in a population of cells in a nonrecurrent manner, thus creating a heterogeneous cell population with a specific chromosomal rearrangement frequency of less than 4% among 50–100 mitosis [46] and may also be represented by clonal chromosomal alterations (CCA), which are recurrent chromosomal alterations that are found at least twice in a population of 20–40 mitosis, or in more than 5% of the cells. Thus, NCCA reflect a more dynamic genome system, and is a better indicator of CIN, while CCA reflects a more stable system [37,42].

Another representative event of CIN is genomic chaos, a massive reorganization of the genome, triggered by one event of cellular crisis leading to chromosome fragmentation with the excess of DSBs generating extreme structural rearrangements such as chromotripsis, defined by multiple rearrangement occurring within a chromosome, whose origin is related to the fragmentation of the chromosomal material in the micronuclei, and chromoplexy, another type of genome chaos consisting in fragmentation and reshuffling of the genetic material among several chromosomes, generating multiple translocations among multiple chromosomes (Figure 1). A cell population may be recognized as having genome chaos when it presents a highly heterogeneous cell population with NCCA, and a number of cells carrying chromotripsis and chromoplexy [47].

NCCA are an indisputable reflection of CIN and karyotypic heterogeneity. In a genomic or karyotypic system that presents genomic instability, each cell may have a different potential for survival and evolution while facing environmental challenges. Thus, by classical mechanisms of natural selection, some chromosomal alterations may become clonal (present in more than 5% of the cell population) and transient. However, some CCAs can be selected and persist if they confer an adaptive advantage, which is generally related to a greater resistance to the adverse environment and to conferring a reproductive advantage; this generates an NCCA/CCA generation cycle that can lead to the formation of a neoplasm [49].

Experimentally, it has been tested in vitro that chemotherapeutic agents such as doxorubicin and Mitomycin-C, induce high chromosomal damage of the NCCA type, such as complex chromosomal alterations, chromotripsis, chromoplexy and other cytogenetic alterations like heterogeneous chromosomal condensation or fragmentation [47]. The experiments of Liu and colleagues have clearly shown that drugs used as anticancer agents induce primarily NCCA and karyotypic chaos, and that this karyotypic heterogeneity is essential for the cell population to survive and adapt to chemical stress, giving rise to more stable karyotypic systems with high survival and reproduction capacity in an adverse environment. This cellular population generated by the stress of anticancer drugs, presents heterogeneous karyotypes accompanied by transcriptome and phenotype heterogeneity. Importantly, these cells retain cellular heterogeneity by maintaining the NCCA through a “fuzzy inheritance”, which is a strong strategy to survive because it has a large chance to produce a large number of potential survivors, most of which are distinctively different [50]. This condition has been linked to the punctuated phase of cancer evolution and, indeed, NCCAs are present in the key transition stages of cancer evolution such as immortalization, transformation, metastasis and drug resistance [49].

## 6. Impact of Anticancer Therapy in Noncancerous Somatic Cells from Patients with Hodgkin Lymphoma

There are several studies showing that CT/RT have a genotoxic effect on somatic cells from HL patients [8,11,27,33,36,51,52,53]. Smith and colleagues [27] detected the genotoxic effects induced by MOPP/RT treatment through painting of chromosome 4 in lymphocytes from patients who were treated 12 to 24 years before the study. They found an increase in chromosomal translocations up to 24 years after treatment, which suggested that anticancer treatment induced permanent damage in the bone marrow hematopoietic cells. In another study, using chromosome banding analysis, it was found a significant increase in chromosome breaks, acentric fragments, dicentrics, and rings in the lymphocytes from HL patients who received MOPP/ABV and RT, which persisted six months after treatment [26]. Similarly, in lymphocytes of HL patients treated with BEACOPP, EBVP, ABVD, and MOPP/ABV, using FISH analysis with painting probes for chromosomes 1, 3, and 4, M’Kacher et al. [51] observed an increase in the frequency of structural chromosomal rearrangements before CT and two years later, a significant increase in complex chromosomal rearrangements involving several breaks and more than two chromosomes [51].

**Figure 2:**
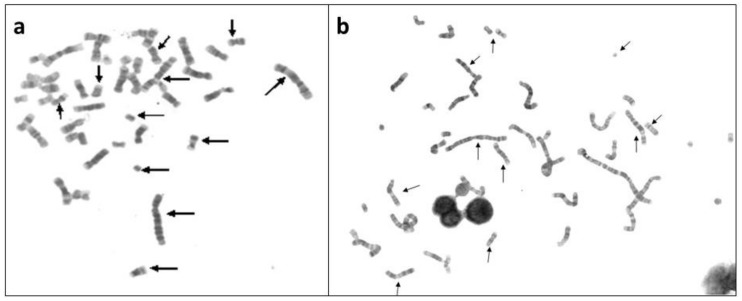
NCCA observed in a group of 20 HL survivors up to 17 years after anticancer treatment MOPP (Mechlorethamine, Oncovin, Procarbazine, and Prednisone), with or without radiotherapy. (**a**) metaphase in peripheral blood lymphocytes from a HL patient 2 years after MOPP treatment and (**b**) metaphase from a HL survivor 13 years after MOPP treatment [33]. Arrows indicate abnormal chromosomes. Note in the interphase nuclei, the chromatin bridges, indicating chromosomal abnormalities that prevented a normal segregation.

The persistence of structural chromosome rearrangements was confirmed by Salas et al. [33], who studied the genotoxic consequences of the MOPP with or without RT in 20 HL patients 2–17 years after the therapy stress. In this study, 1000 lymphocytes in metaphase per patient were analyzed with G banding and 13 out of 20 survivor patients were found to have a high frequency of breaks and NCCA chromosomal structural rearrangements (Figure 2).

Most of the aberrations were nonclonal, with a unique or multiple alterations per cell, consistent with persistent CIN as a result of anticancer treatment. Only one patient showed a CCA structural consisting in a deletion del(17)(p11.2p11.2) in three cells [33]. The majority of chromosomal rearrangements found in this group of HL survivors were NCCA, within a little population of cells with genomic variation that could be detected only because a large number of cells were studied (Figure 2).

**Figure 3:**
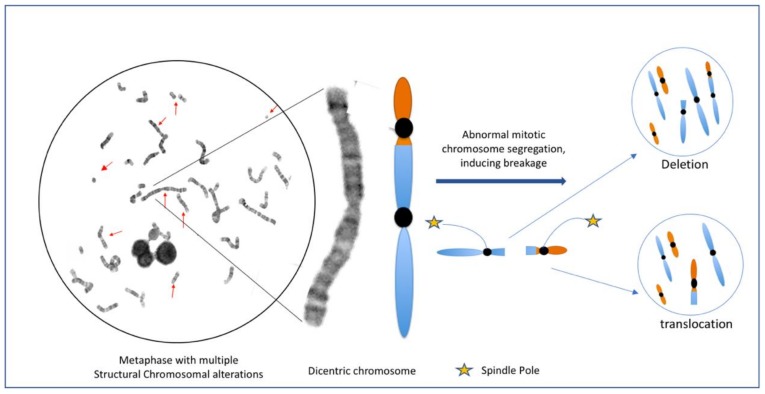
Metaphase of a lymphocyte from a survivor of HL after 13 years post MOPP treatment, with multiple structural NCCA [33]. Note that the possible daughter cells emerge with different alterations such as deletion or translocation, as a result of erroneous mitotic segregation of only one aberrant chromosome. Even when the daughter cells do not share the same structural aberration between them or with the progenitor cell, they may be clonal cells with multiple NCCA and without CCA.

These findings suggest the persistence of a population of hematopoietic stem cells with altered karyotype system due to the stress of the anticancer therapy that maintained their heterogeneous karyotype for up to 17 years after the treatment in 13/20 survivors. It is important to note that in these cells with heterogeneous genomes, cells of a clone do not necessarily show a CCA, due to the complex genome system generating daughter cells that may carry different NCCA resulting in highly dynamic karyotypes (Figure 3) [33]. This population of cells within each patient, is a source of conserved karyotypic heterogeneity that could eventually become a more stable population that acquire CCA and start a pathway towards the progression of cancer. Patients presented with a clonal abnormality, must be carefully followed in the oncology service because may belong to the 10–20% of HL survivors who develop a second cancer.

Currently, ABVD is extensively used with or without RT as anticancer treatment for HL patients because is considered less cytotoxic and genotoxic due to its lower content of alkylating agents and good preservation of sperm production. However, in a longitudinal study with HL patients treated with ABVD/RT, Ramos et al. [48] found that both NCCA and chaotic karyotypes were induced by the stress of this CT and RT in somatic cells, and the damage persisted at least until one year later [48]. The study was performed using multicolor fluorescence in situ hybridization (M-FISH) in metaphases of peripheral blood lymphocytes from patients diagnosed with HL, with sampling times before treatment, during treatment (between second and third cycle of ABVD) and after treatment, one year after the ABVD/RT. The analysis of 50–100 metaphases with M-FISH showed that NCCA consisted of both numerical and structural alterations, with structural NCCA being the most frequent type of chromosomal aberrations.

**Figure 4:**
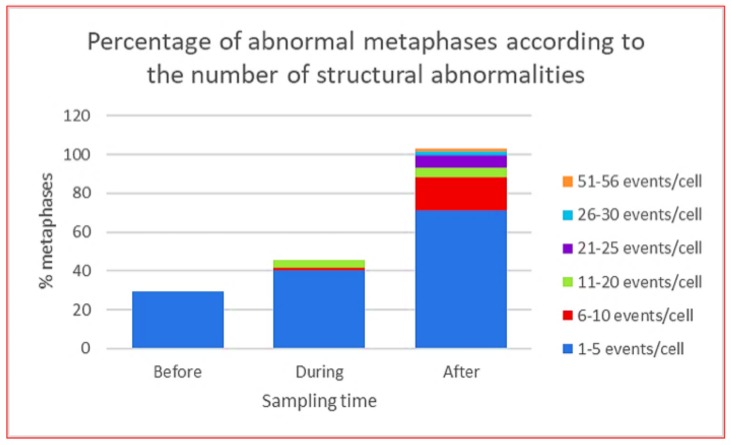
Total population of abnormal metaphases in lymphocytes from HL patients at each indicated sampling time, before, during and after the stress of CT ABVD/RT. The graph shows the percentage of abnormal metaphases according to the number of structural abnormalities per cell [48].

CIN was found in all patients, represented by NCCA involving both numerical and structural abnormalities, however, the highest frequency of damage was structural including simple and complex translocations. In addition, chromosomal chaos was observed one year after treatment indicating that new aberrations were continuously produced in four out of five patients and only in one patient NCCA diminished after one year of treatment. Multiple translocations were found in the same cell, in addition to numerical NCCA. After one year of the anticancer stress, samples presented with a 40-fold increase (*p* < 0.0001; one-tailed Fisher’s exact test) in total abnormalities per cell (0.96 ab/ cell) with respect to control samples (0.024 ab/ cell). Whereas during treatment and before treatment samples showed a nine-fold and four-fold increase (*p* < 0.0001; one-tailed Fisher’s exact test), respectively. The percentage of cells with NCCA was very high in four out of five HL survivors, ranging between 17.7 and 39.1% of abnormal cells with 1–56 abnormalities per cell. It is important to note that before treatment the abnormal cells only presented with 1–5 NCCA/cell, during treatment cells presented with 1–20 NCCA/cell and after treatment aberrant cells presented with 1–56 NCCA/cell, including genomic chaos (Figure 4). CIN and genomic chaos had been referred to as characteristics of cancer cells, however, in this in vivo study, the cells are peripheral blood lymphocytes stimulated with M-phytohemagglutinin, from patients with no evidence of hematological malignancy or relapse of the original cancer [48].

**Figure 5:**
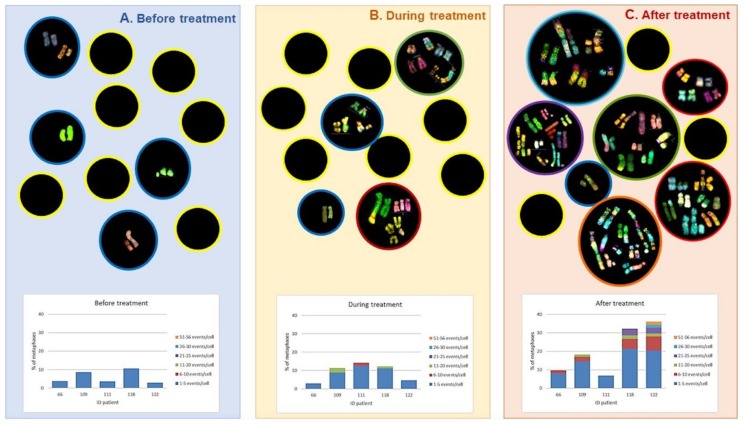
Upper part: M-FISH analysis of peripheral blood lymphocytes in samples from HL patients. Sampling occurred before, during, and one year after completion of ABVD/radiotherapy treatment. Black circles represent cells: cells with yellow outline represent cells with normal karyotypes; cells with colored outlines represent cells with abnormal karyotypes. The graphs in the lower part show the distribution of the abnormal cells according to the number of chromosomal aberrations, for each patient. A. Before treatment, showing alterations in less number and less complexity. B. During treatment, the number of cells with more damage increases even with complex karyotypes. C. After treatment, up to 60% of cells with chromosomal alterations were observed, and with the highest number of aberrations per cell, including chaotic karyotypes [48].

The study of Stephens et al. in 2011 [54] showed clearly that chromothripsis is present in at least 2–3% of all cancers and, in 2014, Liu et al. [47] monitored, in an experimental system consisting of four cell lines, the process of generating genomic chaos, demonstrating that CT agents are able to induce karyotypic heterogeneity in a cell population in vitro, presenting with NCCA and diverse types of damage such as that associated with genomic chaos. This valuable information from in vitro studies or directly in tissue from tumors [47,54], strongly suggested that karyotypic heterogeneity could be produced by anticancer treatment in vivo, in noncancerous cells, and that the population with karyotypic diversity could be the substrate for the evolution toward a new cancer related to treatment, which occur in a high proportion of patients [36]. The results of Ramos et al. [48] showed in vivo that a fraction of normal hematopoietic cells from HL patients respond to the CT/RT stress with a high proportion of NCCA leading to a heterogeneous cell population with complex karyotypes, which is continuously producing mature lymphocytes with NCCA, and demonstrated that diverse karyotypic systems are induced by anticancer treatment, as previously suggested by several authors [37,46,47] (Figure 5).

## 7. Role of Virus Activation in the Generation of Chromosomal Instability in Somatic Noncancerous Cells

A high percentage of the human population is infected by several types of virus, among the most common are EBV, herpes simplex virus 1 and JC polyomavirus (JCV); 40–90% of HL patients are infected by EBV and an even a higher percentage present JCV [2,55]. These viruses are generally in a latent state preserving a balance between the efficiency of the immune system and the copy number of latent viruses; however, many events including aging, chemotherapy, and radiation can cause an increase in the copy number of viruses that leads to an insufficient immune response that results in an increased risk of disease [56]. There is evidence that radiation and some chemotherapy drugs, like doxorubicin and cisplatin, induce direct reactivation of EBV latent infection [57,58].

M’Kacher et al. [55] studied the reactivation of EBV and JCV viruses induced by anticancer treatment in peripheral blood lymphocytes of HL patients. They found that the viral load significantly increased when the anticancer treatment was applied (Standard CT, half of patients CT+RT), indicating the reactivation of both type of viruses. During treatment 55% of HL patients showed “rogue” cells that presented elevated numbers of structural and numerical chromosomal abnormalities. The high copy number of viral sequences was associated with CIN represented by cells with micronuclei or NCCA and viral presence; one year after treatment the viral load decreased to pre-treatment values and rogue polyploid cells did not show viral sequences. Co-activation of EBV and JCV was accompanied by a higher frequency and complexity of chromosomal aberrations, as well as a lower freedom from progression (FFP) [55]. There is little information on the mechanisms by which viral activation can induce CIN; Wu et al. [59] found that early lytic cycle proteins, especially EBV DNase (BGLF5), induce CIN in epithelial cells. On the one hand, the expression of EBV DNase induced DNA breakdown that was visible as DSBs, micronuclei, and chromosomal aberrations; on the other hand, an indirect mechanism was identified, since EBV DNase was also found to repress the expression of some DNA repair genes [59].

The above data allows us to suggest that, in addition to the well-known induction of direct DNA damage by CT and RT, an indirect effect on the generation of NCCA in noncancerous cells is the lytic cycle of latent viruses that generate CIN in the host cell when they are reactivated.

## 8. Impact of Anticancer Therapy on Fertility and Germ Cell Genotoxicity in Patients with Hodgkin Lymphoma

In the past 100 years, semen quality has been declining all around the world. In recent years, in several studies, a 30–40% reduction in the sperm count in healthy men has been reported in several studies [60]. One of the possible causes of this is the use of drugs that impair semen quality. It has been proven that individual chemicals, including those commonly used in CT, induce adverse effects on the quantity and quality of sperm; however, we know very little about the effects of complete CT/RT regimens on the risks of inducing chromosomal abnormalities in the sperm from treated patients [61].

In HL patients, as in other types of cancer, survival is improving as the therapies do, but long-term adverse consequences, including a strong effect on germ cells have been observed. Alkylating agents such as procarbazine are highly toxic to the testis, causing depletion of the germinal epithelium and aplasia of germinal cells [62,63]. Most patients with HL, have poor semen quality following treatment and a low sperm density that is presented as oligospermia and permanent or transient azoospermia [64,65].

Male HL patients who received MOPP as antineoplastic treatment with deleterious effects on spermatogenesis [31] show a significant decrease in semen quality; sperm count is affected, producing azoospermia or severe oligospermia even 22 years after the end of the treatment. Multiple studies have shown that MOPP, COPP, BEACOPP, and ABVD treatments in patients with HL are genotoxic and induce azoospermia at different levels. The most aggressive treatment is MOPP, since 90% to 100% of survivors have azoospermia [15,63,66,67,68]. In contrast, ABVD is less toxic causing transient azoospermia in one-third of patients, and most of them recover sperm production [67,68,69] (Table 2).

The studies on genotoxicity in female germ cells are scarce due to the enormous difficulty of obtaining them in sufficient quantity and quality to perform the analysis. However, there are studies investigating cytotoxicity and fertility. Ovary is not a tissue with great proliferative capacity, it has a fixed number of germ cells produced during fetal life, and they complete their meiotic divisions during puberty, so they are more resistant than the testis to RT [72]. However alkylating agents are very toxic to the female reproductive organ, causing oocyte destruction and follicular depletion leading to ovarian failure and irreversible amenorrhea in a dose-dependent manner. Surviving HL women develop early menopause and absence of pregnancy [2]. Therapies MOPP, BEACOPP, and also CHOP which includes Cyclophosphamide, Doxorubicin (Adriamycin), Vincristine (Oncovin), and Prednisone, are treatments containing high doses of alkylating agents and cause infertility, ovarian failure, and loss of gonadal function. [73]. On the other hand, in women treated with CT without alkylating agents, ovarian failure is rare [74,75]. Female survivors of cancer who maintain fertility are at increased risk of miscarriage and/or premature birth and therefore require counseling and preconception evaluation by treating physicians [76].

The severe reduction of germ cells after CT/RT in HL patients is related with the genotoxicity of the drugs and radiation, which can directly induce DNA damage. The cell response to DNA damage requires DNA repair and, if not successful, cell death. The genotoxicity of CT treatments in germ cells from HL patients has been studied by several groups utilizing diverse methodology. Brandriff et al. [77] were the first to report the genotoxic effect induced by MOPP with and without RT in spermatozoa of HL patients. Using the technique of fusion of human spermatozoa with hamster eggs to obtain and analyze chromosomes, they showed that between 3 to 20 years after receiving CT, sperm presented with 2% of numerical and 7% of structural chromosomal alterations indicating that the chemical agents were capable of damaging the germ stem cells of HL patients. The authors reported that damage “appeared to be not specific for chromosome pairs or regions to be involved in the structural exchanges,” which may be interpreted as NCCA.

Sperm of HL patients treated with NOVP CT were studied with FISH for chromosomes X, Y, and 8 [78] and X, Y, 18, and 21 [71], in samples obtained before, during, and after CT. A transient increase in the frequency of numerical chromosomal alterations was found, with a highest increase during treatment, which decreased three months after treatment.

ABVD treatment also produced a transient high frequency of chromosomal alterations in spermatozoa of HL patients that decreased 3–18 months later [68,79]. In an interesting study, Patassini et al. [80] analyzed the entire genome of 130 single sperms from three HL patients at the end of three months of ABVD CT using aCGH (array Comparative Genome Hybridization) technique, and found that 24% of the sperm carried numerical and structural chromosomal alterations. Specifically, 31 abnormal sperm presented with sex chromosome aneuploidies, 4/131 sperm with XY disomy, 3/131 sperm with XX disomy, 1/131 sperm with sex chromosomes nullisomy, and 23/131 sperm showed gains and losses in different regions of different chromosomes or complex alterations. According to the criteria for determining the type of chromosomal damage, Patassini et al. observed NCCA in these patients [80] (Table 3).

There have been several groups that have studied the genotoxic effect of anticancer therapy in germ cells, and based on to the results presented, it can be concluded that (1) the treatment with MOPP induces long term genotoxic effects, while the treatment with ABVD induces transient genotoxic effects and (2) to observe the type of damage indicating NCCA and genomic chaos, it is necessary to carry out studies that evaluate, either cytogenetically or molecularly, the whole genome, since the methodologies that use specific FISH probes generate information primarily associated with CCA.

It is important to highlight that MOPP contains procarbazine, which is one of the few chemicals that has been shown to affect germline stem cells [31], while other treatments such as ABVD do not contain agents that induce damage in them. This implies that the fraction of stem cell spermatogonia that had sustained genomic damage and survived MOPP therapy, can continuously generate sperm with genomic damage, and thus, HL patients treated with MOPP would have a long-lasting increase in sperm carrying genomic damage. Germline stem cell killing would also result in greatly reduced sperm count and azoospermia until the surviving stem cells begin cycling again. On the other hand, ABVD targets spermatocytes that would also generate sperm with genomic damage. However, once these sperm have been ejaculated there would be no ‘record’ of the exposure making the damage transient because germline stem cells are not affected. In addition, spermatocyte killing would result in a transient reduction in sperm count that would be quickly replenished by the unaffected stem cells.

## 9. Conclusions

The treatment used for cancer patients, specifically HL, includes a series of drugs that have been shown to be cytotoxic and genotoxic and targeting dividing cells both in in vitro systems or in animal models. This strategy has been very efficient in eliminating cancer cells that are in continuous proliferation. However, because these compounds do not target cancer cells specifically, patients who survive cancer have a large number of noncancerous cells in their body that were also affected by anticancer therapy. As a result of this stress, 10–20% of the population of HL survivors develops a new secondary cancer associated to treatment, among other secondary consequences. Studies performed in HL patients before and after treatment, indicate that the noncancerous population of cells that managed to survive, have a variable number of cells highly affected at the genome level, this is probably due to at least two mechanisms: the direct genotoxic action of the chemical and physical agents used as treatment, and the genotoxic effect of latent virus reactivation mediated by the treatment. Both of these mechanisms lead to genomic damage of the NCCA type and increase the frequency of chromosomal aberrations observed in samples obtained during treatment. Some of these cells can persist for a long period of time up to 24 years post-treatment.

Analyzing the results of several studies (Table 3), it can be observed that the anticancer treatments used in patients with HL affect hematopoietic stem cells, so that 1–24 years after treatment, cells with NCCA and even chaotic karyotypes can still be found. In germ cells however, studies in sperm show that depending on the treatment used, the genotoxic damage may be transient or permanent. When the CT used was MOPP, germ cells showed a long-term effect and NCCA was found in HL survivors up to 20 years post-treatment, whereas when the CT did not include procarbazine, the damage was transient indicating that the stem cells had not been affected. All together, these studies show that the stress of anticancer treatment may have different effects depending on the type of stressor agent and on the type of cells.

Regarding the type of damage that has been found in HL patients and survivors, in all cases, the classic karyotypic damage has been detected, such as numerical alterations and structural alterations including deletions, duplications, translocations, dicentrics, rings, etc. However, observations, primarily by Heng and his group [43], have shown that there is a great diversity of genomic alterations, such as chromosomal fragmentation, asynchronous chromosomal condensation, abnormal interphasic figures, chromatin bridges, etc., that have not been monitored and that represent nonclassical genome damage induced by anticancer stress. It is possible that not quantifying this type of alterations, may be the reason for some unexpected data in the study conducted by Ramos et al. [48], where a smaller number of classical cytogenetic alterations were found during treatment, as compared with post-treatment samples. During treatment samples represent the moment when all the cells of the whole organism were under stress, and it is during this moment when chemical and physical agents as well as reactivated viruses induce DNA damage.

According with the in vitro results of Liu et al. [47], the nonclassical genomic damage could be present in a significantly high proportion, and a fraction of the highly rearranged and chaotic karyotypes could be eliminated. Liu et al. [47] found that when treating cells with chemotherapeutic agents such as those used in these patients, doxorubicin (Adriamycin) and alkylating agents, although there is a high frequency of cell death during stress, some of the chaotic genomes can survive and continue to change until the surviving genomes are selected. In fact, the observations made by Ramos et al. [48] and Salas et al. [33] resemble the pattern proposed by the in vitro experiments of Liu et al. [47] in 2014:

(1) The stress of the anticancer treatment induced cell death of the cancerous cells, the treatment is successful to eliminate the cancer. However, genotoxicity and cell death are induced also in noncancerous cells, such as hematopoietic cells; this process can generate a large number of cells with chromosomal fragmentation and some of them can survive.

(2) The large number of DSBs induced by the anticancer treatment must be repaired by homologous recombination, which is error-free and acts only in the postsynthetic phases of late-S and G2; this repair allows a proportion of the cells to repair the DSBs without generating karyotypic changes, although they can carry point mutations. However, an important repair mechanism for these lesions acting throughout the cell cycle is the nonhomologous recombination or NHEJ, both classical or alternative route, and other similar mechanisms, involving abnormal replication [81], that can generate a random rejoining of the chromosomal fragments, producing a karyotypically heterogeneous cell population, with large CIN, leading the formation of highly rearranged genomes and genomic chaos.

It is important to consider that the studies carried out on HL patients [33,48,77], have not documented the phase of chromosomal fragmentation C-Frag that Liu et al. [47] considers a precursor of CIN and genomic chaos. Nevertheless, the finding of karyotypes with a large amount of complex rearrangements and chromoplexy show that there was fragmentation prior to the reshuffling of elements and that multiple DSBs must coexist to be able to form chromoplexy and the observed complex rearrangements (Figure 1, Figure 2 and Figure 5).

(3) During the stress, most of the cells with chaotic genomes tend to die, which may partially explain the low frequency of cells with high NCCA in during treatment samples from patients treated with ABVD [48]. However, the surviving cells generated daughter cells with inherited NCCA (Figure 3), preserving a population with genomic heterogeneity, since in samples post-treatment, one year or more, the alterations observed were NCCA and genomic chaos (Figure 2 and Figure 5). This diversity of genomes is the substrate for the natural selection (punctuated phase of cancer evolution) and over time can generate a clone of cells with more stable karyotypes and in some cases CCA [37,42,47,49]. In the study of Ramos et al. [48], one year after ABVD treatment, the surviving cells from HL patients presented NCCA but not CCA [48], while in the study of Salas et al. [33], only in one HL survivor a CCA that involved alteration of chromosome 17p was found 13 years after the therapy [33]. These data indicate that in vivo, the selection of more stable genomes, with CCA, and possible mutational and epigenetic changes, facilitating the evolution toward a new cancer (Stepwise phase of cancer evolution) may take several years [42,49,82]. This agrees with the information that indicates that second cancer related to therapy in HL survivors appear in approximately 10 years after treatment. These cancer, leukemia, or solid tumors, present with NCCA, CCA, and accumulation of somatic mutations directly associated with anticancer treatment [36,83].

Finally, the behavior of somatic cells is different than that of germ cells in HL patients; hematopoietic stem cells may retain for long time (up to 24 years) a population of cells with a high diversity of karyotypes, NCCA and genomic chaos after several types of CT, while germ stem cells only present long term NCCA after treatment that includes procarbazine (Table 3). Also, the consequences of this long term CIN are very different depending on the cell type; hematopoietic stem cells with NCCA may evolve toward a second cancer related to therapy, while germ stem cells with NCCA tend to disappear leading to oligospermia or azoospermia. According to Heng et al. [37], meiosis acts as a filter of genomic chaos because the reshuffling of segments of chromosomes makes the zygotene phase almost impossible, and most of the cells that survived to the CT stress, will die during meiosis. If stem cell spermatogonia that were exposed to MOPP are eliminated by meiosis, then a majority of these HL survivors are oligospermic or azoospermic (Table 3), preventing the karyotypic heterogeneity at the organism level. Meanwhile, somatic hematopoietic cells do not divide by meiosis, and thus they preserve NCCA and may evolve toward a clone with selective advantage and cancer.

The data presented here resemble the results that Liu et al. [47] obtained in vitro and confirm that in HL patients, genomic chaos is generated by the stress of anticancer therapy, directly by damaging DNA and indirectly by the genotoxicity induced by reactivated viruses, and that a population of hematopoietic stem cells are preserved with great karyotypic heterogeneity and could be in the punctuated phase of evolution toward a second cancer. The fact that 80–90% of surviving HL patients do not develop a second cancer indicates that the environment represented by the whole organism has barriers that are insuperable for most cell populations with NCCA that could evolve towards cancer, but a 10–20% manages to overcome these barriers, showing that evolution acts at the cellular level and in any environment.

It is important to know and understand the mechanisms that lead to the morbidity and mortality that cancer survivors present, since only in this way can new detection and treatment strategies be integrated for managing the secondary consequences of anticancer treatment in HL patients.

## Figures and Tables

**Table 1:** Chemical compounds used in chemotherapy with genotoxic effect on somatic and germinal cells.

Type	Drug	DNA Lesion	Altered Mechanism	Cytogenetic Alterations
Alkylating agents(Monofunctional and Bifunctional)	**Nitrogen mustard (Mechlorethamine)** **Dacarbazine** **Procarbazine** Mitomycin C Cyclophosphamide Ethyl-nitrosourea Melphalan Cisplatin Ifosphamide Clorambucil	Base damage Bulky adducts DNA intrastrand crosslinks DNA interstrand crosslink Double strand breaks	Interferes with DNA synthesis	Chromosomal deletions, insertions, inversions and translocations
Antibiotics	**Doxorubicin (Adriamycin)** Daunorubicin Epirubicin Idarubicin **Bleomycin**	Free radicals Crosslink DNA Single strand breaks Double strand breaks Intercalant of DNA	Blocking of DNA replication and transcription	Chromatid and chromosome-type aberrations, translocations, dicentric, acentric, and other aberrations related to damage of telomere
Mitosis inhibitors	**Vincristine (Oncovin)** **Vinblastine** Vinorelbine	Induce aneuploidy	Interference with tubulin polymerization and inhibits mitotic spindle	Aneuploidy and polyploidy
Topoisomerase II inhibitors	Daunorubicin Epirubicin **Mitoxantrone (Novantrone)** Camptothecin Etoposide	Single strand breaks Double strand breaks Replication lesions	Inhibition of DNA synthesis by forming a complex with Topo II and DNA	Chromosomal translocations, aneuploidy, polyploidy and endoreduplication

In **bold**, the drugs used in CT for HL.

**Table 2:** Consequences of anticancer treatment on the sperm count in HL patients.

Anticancer Treatment	Pre-Treatment	Post-Treatment *			Reference
	Normospermia (% of Patients)	Normospermia (% of Patients)	Oligospermia (% of Patients)	Azoospermia (% of Patients)	
MOPP	100	28	24	48	Da Cunha et al., 1984 [63]
MOPP	100	3	0	97	Viviani et al., 1985 [15]
MOPP	84	0	62	38	Meistrich et al., 1997 [66]
MOPP	100	20	35	45	Sánchez et al., 2008 [70]
MOPP	100	NA	NA	75	Bujan et al., 2014 [67]
ABVD	100	46	21	33	Viviani et al., 1985 [15]
ABVD	100	80	15	5	Sánchez et al., 2008 [70]
ABVD	100	100	0	0	Bujan et al., 2014 [67]
NOVP	100	50	50	0	Frias et al., 2003 [71]

NA = Not available. * The post-treatment time used in the studies was variable, from one month to 23 years.

**Table 3:** Studies on the genotoxic effect of anticancer treatment in HL patients.

Anticancer Therapy	Chromosomal Damage	Reference (Technique)
Lymphocytes		
MOPP/RT 6 cycles	* Chromosomal translocations, NCCA (Persistent up to 24 years)	Smith et al., 1992 [27] (FISH, painting of chromosome 4)
MOPP/ABV 6–9 cycles	* NCCA structural. Chromosome breaks, acentric fragments, dicentrics, and micronucleus (Persistent at six months)	Bilban-Jakopin and Bilban, 2001 [26] (Nonbanded chromosomes)
BEACOPP, EBVP, ABVD, MOPP/ABV RT (combination not specified) 6 cycles	* NCCA structural (Persistent up to 2 years)	M’Kacher et al., 2003 [51] (FISH, Painting of chromosomes 1, 3, and 4)
MOPP/RT 2–9 cycles	NCCA numerical and structural Persistent (up to 17 years)	Salas et al., 2012 [33] (G-Banding Chromosomes)
ABVD/RT 6–8 cycles	NCCA numerical and structural including genomic chaos (Persistent at 1 year)	Ramos et al., 2018 [48] (M-FISH)
**Spermatozoa**		
MOPP/RT 2–6 cycles	* Numerical and structural NCCA (Persistent up to 20 years)	Brandriff et al.,1994 [77] (Nonbanded chromosomes)
CHOP/MOPP/ABV 4–7cycles	Hyperhaploidy, disomy, and diploidy (Persistent; decrease at 2 years)	Martínez et al., 2017 [68] (FISH, specific probes)
NOVP 3 cycles	Disomies, diploidies, and complex genotypes involving the X, Y and 8 chromosomes (Transient; decrease at 3 months)	Robbins et al., 1997 [78] (FISH, specific probes)
NOVP 3 cycles	Disomies, diploidies, and complex genotypes involving the X, Y and 18 and 21 chromosomes (Transient; decrease at 3 months)	Frias et al., 2003 [71] (FISH, specific probes)
ABVD/RT 4–8 cycles	Disomy XY, XX, Nullisomy 13 and 21 (Transient; decrease at 18 months)	Tempest et al., 2008 [79] (FISH, specific probes)
ABVD (number of cycles non-specified)	Disomies XY, XX, Sex chromosome nullisomy, loss and/or gain of part of chromosomes, and complex alterations	Patassini et al., 2013 [80] (microarrays aCGH)
ABVD/RT 4–7 cycles	Hiperhaploidy, disomy, and diploidy (Transient; decrease at 3 months)	Martínez et al., 2017 [68] (FISH, specific probes)

* Authors describe chromosomal abnormalities that are compatible with NCCA, however they do not call it NCCA.

## Supplementary Materials

The following are available online at http://www.mdpi.com/2073-4425/10/1/37/s1. Table S1: Recently used chemotherapy pediatric intermediate in high-risk Hodgkin lymphoma; Table S2: Targeted drugs and several monoclonal antibodies for Hodgkin lymphoma; Table S3: Complexity and types of rearrangements according with the number of co-ocurring DSBs.

## References

[1] Miller K.D., Siegel R.L., Lin C.C., Mariotto A.B., Kramer J.L., Rowland J.H., Stein K.D., Alteri R., Jemal A. (2016). Cancer treatment and survivorship statistics, 2016. CA Cancer J. Clin..

[2] Engert A., Horning S.J. (2015). Hodgkin Lymphoma: A Comprehensive Update on Diagnostics and Clinics.

[3] Ansell S.M. (2015). Hodgkin Lymphoma: Diagnosis and Treatment. Mayo Clin. Proc..

[4] De Fine Licht S., Rugbjerg K., Gudmundsdottir T., Bonnesen T.G., Asdahl P.H., Holmqvist A.S., Madanat-Harjuoja L., Tryggvadottir L., Wesenberg F., Hasle H. (2017). Long-term inpatient disease burden in the Adult Life after Childhood Cancer in Scandinavia (ALiCCS) study: A cohort study of 21,297 childhood cancer survivors. PLoS Med..

[5] Rendón-Macías M.E., Valencia-Ramón E.A., Fajardo-Gutiérrez A., Castro-Ríos A. (2016). Incidence of Childhood Hodgkin Lymphoma in Mexico by Histologic Subtypes and Socioeconomic Regions. J. Pediatr. Hematol. Oncol..

[6] Kaseb H., Babiker H. (2018). Cancer, Lymphoma, Hodgkin.

[7] Weniger M., Barth T., Möller P. (2006). Genomic Alterations in Hodgkin’s Lymphoma. Int. J. Hematol..

[8] Cuceu C., Hempel M.W., Sabatier L., Bosq J., Carde P., M’kacher R. (2018). Chromosomal Instability in Hodgkin Lymphoma: An In-Depth Review and Perspectives. Cancers.

[9] Rotunno M., McMaster M.L., Boland J., Bass S., Zhang X., Burdett L., Hicks B., Ravichandran S., Luke B.T., Yeager M. (2016). Whole exome sequencing in families at high risk for Hodgkin lymphoma: Identification of a predisposing mutation in the KDR gene. Haematologica.

[10] Knecht H., Mai S. (2017). LMP1 and Dynamic Progressive Telomere Dysfunction: A Major Culprit in EBV-Associated Hodgkin’s Lymphoma. Viruses.

[11] M’Kacher R., Bennaceur-Griscelli A., Girinsky T., Koscielny S., Delhommeau F., Dossou J., Violot D., Leclercq E., Courtier M.H., Beron-Gaillard N. (2007). Telomere shortening and associated chromosomal instability in peripheral blood lymphocytes of patients with Hodgkin’s lymphoma prior to any treatment are predictive of second cancers. Int. J. Radiat. Oncol. Biol. Phys..

[12] Fox M., Scott D. (1980). The genetic toxicology of nitrogen and sulphur mustard. Mutat. Res. Genet. Toxicol..

[13] Povirk L.F., Shuker D.E. (1994). DNA damage and mutagenesis induced by nitrogen mustards. Mutat. Res. Genet. Toxicol..

[14] Hagemeister F., Purugganan R., Fuller L., McLaughlin P., Swan F.J., Romaguera J., Rodriguez M., Cabanillas F. (1995). Treatment of early stages of Hodgkin’s disease with novantrone, vincristine, vinblastine, prednisone, and radiotherapy. Semin Hematol..

[15] Viviani S., Santoro A., Ragni G., Bonfante V., Bestetti O., Bonadonna G. (1985). Gonadal toxicity after combination chemotherapy for hodgkins disease comparative results of MOPP vs ABVD. Eur. J. Cancer Clin. Oncol..

[16] Jain S., Kapoor G., Bajpai R. (2016). ABVD-Based Therapy for Hodgkin Lymphoma in Children and Adolescents: Lessons Learnt in a Tertiary Care Oncology Center in a Developing Country. Pediatr. Blood Cancer.

[17] Kelly K.M. (2015). Hodgkin lymphoma in children and adolescents: Improving the therapeutic index. Blood.

[18] Behringer K., Josting A., Schiller P., Eich H.T., Bredenfeld H., Diehl V., Engert A. (2004). Solid tumors in patients treated for Hodgkin’s disease: A report from the German Hodgkin Lymphoma Study Group. Ann. Oncol..

[19] Küppers R., Engert A., Hansmann M.-L. (2012). Hodgkin lymphoma. J. Clin. Investig..

[20] Journy N., Mansouri I., Allodji R.S., Demoor-Goldschmidt C., Ghazi D., Haddy N., Rubino C., Veres C., Zrafi W.S., Rivera S. (2018). Volume effects of radiotherapy on the risk of second primary cancers: A systematic review of clinical and epidemiological studies. Radiother. Oncol..

[21] Kaplan H. (1962). The radical radiotherapy of regionally localized Hodgkin’s disease. Radiology.

[22] Yeoh K.W., Mikhaeel N. (2010). Role of Radiotherapy in Modern Treatment of Hodgkin’s Lymphoma. Adv. Hematol..

[23] Yahalom J. (2009). Does radiotherapy still have a place in Hodgkin lymphoma?. Curr. Hematol. Malig. Rep..

[24] Prosnitz L.R. (1976). Radiation doses following intensive chemotherapy in the treatment of hodgkin’s disease. Int. J. Radiat. Oncol..

[25] Filippi A.R., Franco P., Ciammella P. (2012). Role of modern radiation therapy in early stage Hodgkin’s lymphoma: A young radiation oncologists’ perspective. Rep. Pract. Oncol. Radiother..

[26] Bilban-Jakopin C., Bilban M. (2001). Genotoxic effects of radiotherapy and chemotherapy on circulating lymphocytes in patients with Hodgkin’s disease. Mutat. Res. Toxicol. Environ. Mutagen..

[27] Smith L.M., Evans J.W., Mori M., Brown J.M., Phil D. (1992). The frequency of translocations after treatment for Hodgkin’s disease. Int. J. Radiat. Oncol. Biol. Phys..

[28] Marín A., Martín M., Liñán O., Alvarenga F., López M., Fernández L., Büchser D., Cerezo L. (2014). Bystander effects and radiotherapy. Reports Pract. Oncol. Radiother..

[29] Venkatesan S., Natarajan A.T., Hande M.P. (2015). Chromosomal instability—Mechanisms and consequences. Mutat. Res. Genet. Toxicol. Environ. Mutagen..

[30] Marcu L.G. (2017). Photons–Radiobiological issues related to the risk of second malignancies. Phys. Med. Eur. J. Med. Phys..

[31] Witt K.L., Bishop J.B. (1996). Mutagenicity of anticancer drugs in mammalian germ cells. Mutat. Res. Mol. Mech. Mutagen..

[32] Lowenthal R.M., Eaton K. (1996). Toxicity of chemotherapy. Hematol. Oncol. Clin. N. Am..

[33] Salas C., Niembro A., Lozano V., Gallardo E., Molina B., Sanchez S., Ramos S., Carnevale A., Perez-Vera P., Rivera-Luna R. (2012). Persistent genomic instability in peripheral blood lymphocytes from hodgkin lymphoma survivors. Environ. Mol. Mutagen..

[34] Van Eggermond A.M., Schaapveld M., Janus C.P., de Boer J.P., Krol A.D., Zijlstra J.M., van der Maazen R.W., Kremer L.C., van Leerdam M.E., Louwman M.W. (2017). Infradiaphragmatic irradiation and high procarbazine doses increase colorectal cancer risk in Hodgkin lymphoma survivors. Br. J. Cancer.

[35] Van Leeuwen F.E., Ng A.K. (2016). Long-term risk of second malignancy and cardiovascular disease after Hodgkin lymphoma treatment. Hematol. Am. Soc. Hematol. Educ. Progr..

[36] Salas C., Perez-Vera P., Frias S. (2011). Genetic abnormalities in leukemia secondary to treatment in patients with Hodgkin’s disease. Rev. Investig. Clin. Transl. Investig..

[37] Heng H.H., Bremer S.W., Stevens J.B., Horne S.D., Liu G., Abdallah B.Y., Ye K.J., Ye C.J. (2013). Chromosomal instability (CIN): What it is and why it is crucial to cancer evolution. Cancer Metastasis Rev..

[38] García-Castillo H., Vásquez-Velásquez A.I., Rivera H., Barros-Núñez P. (2008). Clinical and genetic heterogeneity in patients with mosaic variegated aneuploidy: Delineation of clinical subtypes. Am. J. Med. Genet. Part A.

[39] Rodríguez A., D’Andrea A. (2017). Fanconi anemia pathway. Curr. Biol..

[40] García-De Teresa B., Hernández-Gómez M., Frías S. (2017). DNA Damage as a Driver for Growth Delay: Chromosome Instability Syndromes with Intrauterine Growth Retardation. BioMed Res. Int..

[41] Rao C.V., Asch A.S., Yamada H.Y. (2017). Emerging links among Chromosome Instability (CIN), cancer, and aging. Mol. Carcinog..

[42] Rangel N., Forero-Castro M., Rondon-Lagos M. (2017). New Insights in the Cytogenetic Practice: Karyotypic Chaos, Non-Clonal Chromosomal Alterations and Chromosomal Instability in Human Cancer and Therapy Response. Genes.

[43] Heng H.H., Liu G., Stevens J.B., Abdallah B.Y., Horne S.D., Ye K.J., Bremer S.W., Chowdhury S.K., Ye C.J. (2013). Karyotype Heterogeneity and Unclassified Chromosomal Abnormalities. Cytogenet. Genome Res..

[44] Bohlander S.K., Kakadia P.M., Michael G.B., Ried T. (2015). Chromosomal Instability in Cancer Cells.

[45] Giam M., Rancati G. (2015). Aneuploidy and chromosomal instability in cancer: A jackpot to chaos. Cell Div..

[46] Heng H.H.Q., Liu G., Bremer S., Ye K.J., Stevens J., Ye C.J. (2006). Clonal and non-clonal chromosome aberrations and genome variation and aberration. Genome.

[47] Liu G., Stevens J.B., Horne S.D., Abdallah B.Y., Ye K.J., Bremer S.W., Ye C.J., Chen D.J., Heng H.H. (2014). Genome chaos Survival strategy during crisis. Cell Cycle.

[48] Ramos S., Navarrete-Meneses P., Molina B., Cervantes-Barragán D.E., Lozano V., Gallardo E., Marchetti F., Frias S. (2018). Genomic chaos in peripheral blood lymphocytes of Hodgkin’s lymphoma patients one year after ABVD chemotherapy/radiotherapy. Environ. Mol. Mutagen..

[49] Heng H., Regan S.M., Liu G., Ye C.J. (2016). Why it is crucial to analyze non clonal chromosome aberrations or NCCAs?. Mol. Cytogenet..

[50] Ye C.J., Regan S., Liu G., Alemara S., Heng H.H. (2018). Understanding aneuploidy in cancer through the lens of system inheritance, fuzzy inheritance and emergence of new genome systems. Mol. Cytogenet..

[51] M’Kacher R., Girinsky T., Koscielny S., Dossou J., Violot D., Beron-Gaillard N., Ribrag V., Bourhis J., Bernheim A., Paramentier C. (2003). Baseline and treatment-induced chromosomal abnormalities in peripheral blood lymphocytes of Hodgkin’s lymphoma patients. Int. J. Radiat. Oncol. Biol. Phys..

[52] De Mesa R.L., Sierrasesumaga L., Calasanz M.J., de Cerain A.L., Patino-Garcia A. (2000). Nonclonal chromosomal aberrations induced by anti-tumoral regimens in childhood cancer: Relationship with cancer-related genes and fragile sites. Cancer Genet. Cytogenet..

[53] Ryabchenco N., Nasonova V., Antoschina M., Fesenko E., Kondrashova T., Ivanova T., Pavlov V., Ryabikhina N., Terekhova A. (2003). Persistence of chromosome aberrations in peripheral lymphocytes from Hodgkin’s lymphoma remission patients. Int. J. Radiat. Biol..

[54] Stephens P.J., Greenman C.D., Fu B., Yang F., Bignell G.R., Mudie L.J., Pleasance E.D., Lau K.W., Beare D., Stebbings L.A. (2011). Massive genomic rearrangement acquired in a single catastrophic event during cancer development. Cell.

[55] M’kacher R., Andreoletti L., Flamant S., Milliat F., Girinsky T., Dossou J., Violot D., Assaf E., Clausse B., Koscielny S. (2010). JC human polyomavirus is associated to chromosomal instability in peripheral blood lymphocytes of Hodgkin’s lymphoma patients and poor clinical outcome. Ann. Oncol..

[56] Polansky H., Schwab H. (2018). Latent viruses can cause disease by disrupting the competition for the limiting factor p300/CBP. Cell. Mol. Biol. Lett..

[57] Nandakumar A., Uwatoko F., Yamamoto M., Tomita K., Majima H.J., Akiba S., Koriyama C. (2017). Radiation-induced Epstein–Barr virus reactivation in gastric cancer cells with latent EBV infection. Tumor Biol..

[58] Lima R.T., Seca H., Brás S., Nascimento M.S.J., Vasconcelos M.H. (2011). Treatment of Akata EBV-Positive Cells with Doxorubicin Causes More EBV Reactivation than Treatment with Etoposide. Chemotherapy.

[59] Wu C.-C., Liu M.-T., Chang Y.-T., Fang C.-Y., Chou S.-P., Liao H.-W., Kuo K.-L., Hsu S.-L., Chen Y.-R., Wang P.-W. (2010). Epstein-Barr virus DNase (BGLF5) induces genomic instability in human epithelial cells. Nucleic Acids Res..

[60] Sengupta P., Dutta S., Krajewska-Kulak E. (2017). The Disappearing Sperms: Analysis of Reports Published Between 1980 and 2015. Am. J. Mens. Health.

[61] Wyrobek A.J., Schmid T.E., Marchetti F. (2005). Relative Susceptibilities of Male Germ Cells to Genetic Defects Induced by Cancer Chemotherapies. JNCI Monogr..

[62] Byrne J., Mulvihill J.J., Myers M.H., Connelly R.R., Naughton M.D., Krauss M.R., Steinhorn S.C., Hassinger D.D., Austin D.F., Bragg K. (1987). Effects of Treatment on Fertility in Long-Term Survivors of Childhood or Adolescent Cancer. N. Engl. J. Med..

[63] Da Cunha M.F., Meistrich M.L., Fuller L.M., Cundiff J.H., Hagemeister F.B., Velasquez W.S., McLaughlin P., Riggs S.A., Cabanillas F.F., Salvador P.G. (1984). Recovery of spermatogenesis after treatment for Hodgkin’s disease: Limiting dose of MOPP chemotherapy. J. Clin. Oncol..

[64] Anselmo A.P., Cartoni C., Bellantuono P., Maurizi-Enrici R., Aboulkair N., Ermini M. (1990). Risk of infertility in patients with Hodgkin’s disease treated with ABVD vs MOPP vs ABVD/MOPP. Haematologica.

[65] Marmor D., Duyck F. (1995). Male reproductive potential after MOPP therapy for Hodgkin’s disease: A long-term survey. Andrologia.

[66] Meistrich M.L., Wilson G., Mathur K., Fuller L.M., Rodriguez M.A., McLaughlin P., Romaguera J.E., Cabanillas F.F., Ha C.S., Lipshultz L.I. (1997). Rapid recovery of spermatogenesis after mitoxantrone, vincristine, vinblastine, and prednisone chemotherapy for Hodgkin’s disease. J. Clin. Oncol..

[67] Bujan L., Walschaerts M., Brugnon F., Daudin M., Berthaut I., Auger J., Saias J., Szerman E., Moinard N., Rives N. (2014). Impact of lymphoma treatments on spermatogenesis and sperm deoxyribonucleic acid: A multicenter prospective study from the CECOS network. Fertil. Steril..

[68] Martinez G., Walschaerts M., Le Mitouard M., Borye R., Thomas C., Auger J., Berthaut I., Brugnon F., Daudin M., Moinard N. (2017). Impact of Hodgkin or non-Hodgkin lymphoma and their treatments on sperm aneuploidy: A prospective study by the French CECOS network. Fertil. Steril..

[69] Canellos G.P., Anderson J.R., Propert K.J., Nissen N., Cooper M.R., Henderson E.S., Green M.R., Gottlieb A., Peterson B.A. (1992). Chemotherapy of Advanced Hodgkin’s Disease with MOPP, ABVD, or MOPP Alternating with ABVD. N. Engl. J. Med..

[70] Sánchez S., Molina B., Niembro A., Rivera-Luna R., Frias G., Carnevale A., Ulloa-Aguirre A., Badillo D., Cervantes D., Lozano V. (2008). Evaluación de la función gonadal en pacientes con enfermedad de Hodgkin tratados con ABVD o MOPP. Proceedings of the XIII Encuentro Nacional de Investigadores en Salud. Comisión Coordinadora de Institutos Nacionales de Salud y Hospitales de Alta Especialidad.

[71] Frias S., Van Hummelen P., Meistrich M.L., Lowe X.R., Hagemeister F.B., Shelby M.D., Bishop J.B., Wyrobek A.J. (2003). NOVP Chemotherapy for Hodgkin’s Disease Transiently Induces Sperm Aneuploidies Associated with the Major Clinical Aneuploidy Syndromes Involving Chromosomes X, Y, 18, and 21. Cancer Res..

[72] Longo D. (2003). Radiation therapy in the treatment of Hodgkin’s disease—Do you see what I see?. J. Natl. Cancer Inst..

[73] Blumenfeld Z., Avivi I., Eckman A., Epelbaum R., Rowe J.M., Dann E.J. (2008). Gonadotropin-releasing hormone agonist decreases chemotherapy-induced gonadotoxicity and premature ovarian failure in young female patients with Hodgkin lymphoma. Fertil. Steril..

[74] Behringer K., Thielen I., Mueller H., Goergen H., Eibl A.D., Rosenbrock J., Halbsguth T., Eichenauer D.A., Fuchs M., Reiners K.S. (2012). Fertility and gonadal function in female survivors after treatment of early unfavorable Hodgkin lymphoma (HL) within the German Hodgkin Study Group HD14 trial. Ann. Oncol..

[75] Haukvik U.K.H., Dieset I., Bjøro T., Holte H., Fosså S.D. (2006). Treatment-related premature ovarian failure as a long-term complication after Hodgkin’s lymphoma. Ann. Oncol..

[76] Van Dorp W., Haupt R., Anderson R.A., Mulder R.L., van den Heuvel-Eibrink M.M., van Dulmen-den Broeder E., Su H.I., Falck Winther J., Hudson M.M., Levine J.M. (2018). Reproductive Function and Outcomes in Female Survivors of Childhood, Adolescent, and Young Adult Cancer: A Review. J. Clin. Oncol..

[77] Brandriff B.F., Meistrich M.L., Gordon L.A., Carrano A.V., Liang J.C. (1994). Chromosomal damage in sperm of patients surviving Hodgkin’s disease following MOPP (nitrogen mustard, vincristine, procarbazine, and prednisone) therapy with and without radiotherapy. Hum. Genet..

[78] Robbins W.A., Meistrich M.L., Moore D., Hagemeister F.B., Weier H.-U., Cassel M.J., Wilson G., Eskenazi B., Wyrobek A.J. (1997). Chemotherapy induces transient sex chromosomal and autosomal aneuploidy in human sperm. Nat. Genet..

[79] Tempest H.G., Ko E., Chan P., Robaire B., Rademaker A., Martin R.H. (2008). Sperm aneuploidy frequencies analysed before and after chemotherapy in testicular cancer and Hodgkin’s lymphoma patients. Hum. Reprod..

[80] Patassini C., Garolla A., Bottacin A., Menegazzo M., Speltra E., Foresta C., Ferlin A. (2013). Molecular Karyotyping of Human Single Sperm by Array-Comparative Genomic Hybridization. PLoS ONE.

[81] Poot M. (2017). Of Simple and Complex Genome Rearrangements, Chromothripsis, Chromoanasynthesis, and Chromosome Chaos. Mol. Syndromol..

[82] Horne S.D., Pollick S.A., Heng H.H.Q. (2015). Evolutionary mechanism unifies the hallmarks of cancer. Int. J. Cancer.

[83] Rigter L.S., Snaebjornsson P., Rosenberg E.H., Atmodimedjo P.N., Aleman B.M., ten Hoeve J., Geurts-Giele W.R., van Ravesteyn T.W., Hoeksel J., Meijer G.A. (2018). Double somatic mutations in mismatch repair genes are frequent in colorectal cancer after Hodgkins lymphoma treatment. Gut.

