# Supplementary material for: Nonclonal Chromosome Aberrations and Genome Chaos in Somatic and Germ Cells from Patients and Survivors of Hodgkin Lymphoma
Source: Genes (Basel). 2019 Jan 10;10(1):37. doi: 10.3390/genes10010037 (PMC6357137; doi:10.3390/genes10010037)
Supplement: Supplementary file 1 [file genes-10-00037-s001.pdf]

Table S1. Recently used chemotherapy pediatric intermediate in high-risk Hodgkin lymphoma.

Scheme	Compounds	Study	Risk group	Rx	Radiation (Gy)
VBVP	Vinblastine, Bleomycin, Etoposide, Prednisone	French Society of Pediatric Oncology (MDH-90)	IA, IB, IIA, IIB	VBVP 34 (*OPPA 31-2 if PR after cycle 4)	20-40, IF
VAMP	Vinblastine, Doxorubicin, Methotrexate, Prednisone	Stanford, Dana Farber, and St Jude Consortium9	A, IB, IIA, IIB no bulk, no E<br><br>IA, IIA, < 3 nodal sites, no bulk, no E	VAMP 34<br><br>VAMP 34	15-22.5, IF<br><br>CR after 2 cycles: no RT; PR after cycle 2: 25.5 IF
DBVE	Doxorubicin, Bleomycin, Vincristine Etoposide	Children's Oncology Group (POG9426)	IA, IIA, IIIA (no bulk)	DBVE 32-4 (based on response after cycle 2)	vs none; PR: 21, IF 25.5, IF
OEPA	Vincristine, Etoposide, Prednisone, Doxorubicin	German Society of Pediatric Oncology (GPOH7)	IA, IB, IIA	OEPA (males)	CR after cycle 2: no RT; PR after cycle 2: 20-30, IF
OPPA	Vincristine, Prednisone, Procarbazine, Doxorubicin			OPPA (females) 32	
ABVE-PC	Doxorubicin, Bleomycin, Vincristine, Etoposide, Prednisone, Cyclophosphamide	Children's Oncology Group (AHOD0031)	IB, IA/IIA with bulk, IIB, IIIA, IVA	ABVE-PC 34	RER, CR: None
DECA	Dexamethasone, Etoposide, Cisplatin, Cytarabine			SER: 6 DECA 32	RER, CR: 21, IF SER: 21, IF

*Adverse features: hilar disease, bulk, ≥ 4 nodal regions, mediastinal bulk. IF, Involved fields. CR, complete response. PR, partial response. RER, rapid early responder. SER, slow early responder. Modified from Kelly, 2015 [1].

Table S2. Targeted drugs and several monoclonal antibodies for Hodgkin lymphoma.

Drug Type		Drug name	HL Phase	Study
Antibody	Anti-CD30 monoclonal	Brentuximab vedotin	I-III	Younes A. et al., 2010 [2]<br>Younes A. et al., 2012 [3]
	Anti-CD16/CD30 bispecific	AFM13	I	Affimed Therapeutics AG., 2012 [4]
	Anti-CD20 monoclonal antibody	Ofatumumab (ARZERRA)	II	Grupo Español De Linfomas Y Transpalante De Médula Ósea. 2010 [5]
Molecules	Immunomodulatory drug	Lenalidomide (Revlimid)	II	Böll, B., et al., 2010 [6]
	HDAC inhibitor	Resminostat (4SC-201)	I	4SC AG., 2012 [7]
	HDAC inhibitor	4SC-202	I	4SC AG., 2012 [8]
	mTOR inhibitor	Everolimus (RAD001; Affinitor)	II	Johnston, P.B. *et al.*, 2010 [9]

Modified from Küppers *et al.* 2012 [10]

Table S3. Complexity and types of rearrangements according with the number of co-ocurring DSBs

Number of DSBs	Location of the DSBs	Possible Rearrangements
2	DSBs occurs in the same arm of the same chromosome	a) Deletion + ring or + acentric fragment<br>b) Paracentric inversion
	DSBs occurs in the different arms of the same chromosome	c) Ring with deletion + acentric fragment<br>d) Pericentric inversion
	DSBs occur in the same arm of two homologous chromosomes	e) Translocation, interstitial duplications and deletions<br>f) Dicentric chromosome with deletion + acentric fragment
	DSBs occur on different, nonhomologous chromosomes	g) Balanced translocation<br>h) Dicentric chromosome + acentric fragment
3	All the anterior with two DSBs (a-h)	i) All the anterior with two DSBs (a-h) + one deletion or + one inversion
	DSBs occur on three different non-homologous chromosomes	j) Complex three-way balanced translocation<br>k) Dicentric + deletion + acentric fragment

	Two DSBs on the same chromosome and one DSB on a different chromosome	l) Inversion + deletion + acentric fragment<br>m) Interchromosomal insertion
Multiple DSBs	DSBs occur in the same chromosome	n) Chromotripsis, genomic chaos
	DSBs occur in two different chromosomes	o) Chromotripsis, genomic chaos
	DSBs occur in multiple different chromosomes	p) Chromoplexy, complex chromosome rearrangements, multiple deletions and duplications, genomic chaos

References

1. Kelly, K.M. Hodgkin lymphoma in children and adolescents: Improving the therapeutic index. *Blood* **2015**, *126*, 2452–2458.
2. Younes, A.; Bartlett, N.L.; Leonard, J.P.; Kennedy, D.A.; Lynch, C.M.; Sievers, E.L.; Forero-Torres, A. Brentuximab Vedotin (SGN-35) for Relapsed CD30-Positive Lymphomas. *N. Engl. J. Med.* **2010**, *363*, 1812–1821, doi:10.1056/NEJMoa1002965.
3. Younes, A.; Gopal, A.K.; Smith, S.E.; Ansell, S.M.; Rosenblatt, J.D.; Savage, K.J.; Ramchandren, R.; Bartlett, N.L.; Cheson, B.D.; de Vos, S.; et al. Results of a pivotal phase II study of brentuximab vedotin for patients with relapsed or refractory Hodgkin's lymphoma. *J. Clin. Oncol.* **2012**, *30*, 2183–2189, doi:10.1200/JCO.2011.38.0410.
4. Affimed Therapeutics., A. A Study to Assess AFM13 in Patients With Hodgkin Lymphoma. NIH. Clin. 10/1/2010-11/1/2012 2012, Updated 26 June 2011.
5. Grupo Español de Linfomas y Transplante Autólogo de Médula Ósea. Study of Ofatumumab and ESHAP for the Treatment of Hodgkin's Lymphoma.. https://clinicaltrials.gov/ct2/show/NCT01195766
6. Böll, B.; Borchmann, P.; Topp, M.S.; Hänel, M.; Reiners, K.S.; Engert, A.; Naumann, R. Lenalidomide in patients with refractory or multiple relapsed Hodgkin lymphoma. *Br. J. Haematol.* **2010**, *148*, 480–482, doi:10.1111/j.1365-2141.2009.07963.x.
7. 4SC AG. Resminostat (4SC-201) in Relapsed or Refractory Hodgkin's Lymphoma (SAPHIRE). NIH Web Site. Available online: http//clinicaltrials.gov/ct2/show/ NCT01037478.
8. 4SC AG. Oral Histone Deacetylase Inhibitor 4SC-202 in Patients With Advanced Hematologic Malignancies (TOPAS). NIH Web Site. Available online: http//clinicaltrials. gov/ct2/show/NCT01344707.
9. Johnston, P.B.; Inwards, D.J.; Colgan, J.P.; Laplant, B.R.; Kabat, B.F.; Habermann, T.M.; Micallef, I.N.; Porrata, L.F.; Ansell, S.M.; Reeder, C.B.; et al. A Phase II trial of the oral mTOR inhibitor everolimus in relapsed Hodgkin lymphoma. Am. J. Hematol. 2010, 85, 320–324, doi:10.1002/ajh.21664.
10. Küppers, R.; Engert, A.; Hansmann, M.-L. Hodgkin lymphoma. *J. Clin. Investig.* **2012**, *122*, 3439–3447, doi:10.1172/JCI61245.
